# Baoyuan Jiedu Decoction Alleviates Cancer-Induced Myotube Atrophy by Regulating Mitochondrial Dynamics Through p38 MAPK/PGC-1α Signaling Pathway

**DOI:** 10.3389/fonc.2020.523577

**Published:** 2020-09-30

**Authors:** Delong Wang, Weiqiao Chen, Qianyu Bi, Xin Zong, Jiazhao Ruan, Xiangjun Yin, Jixin Wang, Honghua Zhang, Xuming Ji

**Affiliations:** ^1^School of Basic Medical Science, Zhejiang Chinese Medical University, Zhejiang, China; ^2^College of Traditional Chinese Medicine, Shandong University of Traditional Chinese Medicine, Shandong, China; ^3^Zhejiang University-University of Edinburgh Institute, Zhejiang University, Zhejiang, China; ^4^Medical College, Hangzhou Normal University, Zhejiang, China

**Keywords:** cancer cachexia, Baoyuan Jiedu decoction (BJD), traditional Chinese medicine, mitochondrial dynamics, p38 MAPK/PGC-1α pathway, *in vitro*, *in vivo*

## Abstract

Cancer cachexia is a multifactorial syndrome characterized by continuous body wasting and loss of skeletal muscle. Impaired mitochondria function is closely associated with muscle atrophy in cancer cachexia. Our previous study confirmed the effectiveness of Baoyuan Jiedu decoction (BJD) in inhibiting cancer-induced muscle atrophy in an *in vivo* model. However, little is known about its mechanisms in regulating mitochondria dysfunction. In this study, we evaluated the therapeutic effect and action mechanisms of BJD against atrophy both in the Lewis-conditioned medium induced C2C12 myotube atrophy model and in a BALB/c mice xenograft model using mouse colon cancer C26 cells. The mitochondrial content was tested by 10-Non-ylacridine orange staining. Expressions of related proteins and mRNAs were detected by western blotting (WB) and qPCR, respectively. As a result, 18 major components were identified in BJD by ultra-high performance liquid chromatography-quadrupole (UHPLC-Q) Exactive analysis. As shown in the *in vitro* results, BJD treatment prevented prominent myotube atrophy and increased the myotube diameter of C2C12 cells. Besides, BJD treatment increased mitochondrial content and ATPase activity. Furthermore, the protein and mRNA expressions that were related to mitochondrial functions and generation such as cytochrome-c oxidase IV, Cytochrome C, nuclear respiratory factor 1, and mitochondrial transcription factor A were significantly increased in BJD treatment compared to the control group. The *in vivo* results showed that BJD treatment prevented body weight loss and improved the gastrocnemius index in cachexia mice. Moreover, the expressions of Atrogin-1 and muscle RING-finger protein-1 were decreased by BJD treatment. Mechanically, BJD increased the expression of peroxisome proliferator-activated receptor-gamma coactivator 1, and consistently, inhibited the expression of p38 MAPK and its phosphorylation both *in vivo* and *in vitro*. Taken together, this study identified that BJD effectively relieved cancer-induced myotube atrophy and provided a potential mechanism for BJD in regulating mitochondrial dynamics through p38 MAPK/PGC-1α signaling pathway.

## Introduction

Cancer cachexia is a leading cause of morbidity and mortality, resulting in approximately 80% of cancer cases and 40% of cancer-related deaths (1). An ongoing loss of body weight including skeletal muscle mass is the characteristic of cancer cachexia, which lacks effective nutritional support (2, 3). However, there are no therapeutic drugs specific for it. Therefore, focusing on developing effective approaches to prevent cachexia is imperative (4).

The pathogenesis of cancer cachexia is a complex process that is involved in several systemic metabolic disorders, such as anemia, insulin resistance, chronic inflammation, and skeletal muscle protein degradation (5–7). Inflammatory cytokines, typically like TNF-α and IL-6, can increase protein degradation through activation of the NF-κB and the ubiquitin-proteasome pathway (UPP) pathway (8–10). Previous studies showed that muscle atrophy F-Box (MAFbx)/atrogin-1 and muscle RING-finger protein-1 (MuRF-1), two muscle-specific E3 ubiquitin ligases of UPP, were overexpressed in skeletal muscle under cancer-induced muscle atrophy (11). Importantly, UPP-mediated mitochondrial function plays an important role in cancer-induced skeletal muscle atrophy (12). Uncoupling proteins, such as uncoupling protein-2 (UCP2) and uncoupling protein-3 (UCP3), could disrupt the external and internal mitochondrial membrane so that reduce the mitochondrial ATP synthesis for muscle energy metabolism (13).

The normal mitochondrial function is critical to the maintenance of skeletal muscle energy metabolism (14, 15). However, prior studies have noted that the mitochondrial DNA (mtDNA) related with ATP synthesis is significantly reduced and the expression of cytochrome-c oxidase IV (COXIV) is down-regulated in cancer cachectic mice (16–18). This pathogenesis is mainly triggered by increased expression of UCP-2 and UCP-3 (13, 19). Notably, peroxisome proliferator-activated receptor-gamma coactivator 1 (PGC-1α) is a transcriptional coactivator that is responsible for the mitochondrial generation (20). Proliferator-activated receptor-gamma coactivator 1 promotes the expression of nuclear respiratory factor 1 (NRF-1) and mitochondrial transcription factor A (TFAM), which regulates the transcription of mitochondrial genomes (20). Furthermore, PGC-1α also inhibits the induction of Atrogin-1 expression. However, PGC-1α activities can be impaired by many events, including p38 MAPK and AMPK pathway activations, acetylation by the longevity gene SIRT1, and methylation (21–24). p38 MAPK is most abundantly expressed in skeletal muscle and regulates skeletal muscle mass and myotube differentiation (25). It is indicated that p38 MAPK can induce the expression of E3 ligases, including Atrogin-1 and MuRf-1, by targeting the activation of UPP in gastrocnemius muscle (26). Furthermore, a major effect triggered by p38 MAPK during the process of mitochondrial dysfunction is the inhibition of PGC-1α activation (27). Therefore, to improve cancer cachexia treatment, the ways of regulating p38 MAPK/PGC-1α pathway should be figured out.

Traditional Chinese Medicine (TCM) has been widely used in clinical practice for thousands of years in China. Numerous studies have indicated that TCM had important anti-tumor effects (28, 29). Baoyuan Jiedu decoction (BJD), a classical traditional Chinese herbal formula, is used to treat cancer cachexia. Our previous study demostrated that BJD ameliorated cancer-induced myotube atrophy in *Apc*^Min/+^ cachectic mice (30, 31). Furthermore, BJD has been proven to suppress the expression of mitochondria-related UCP2 and UCP3 via inhibiting UPP *in vitro* (32). Due to the close relationship between UPP and mitochondria function, it is proposed that BJD could regulate mitochondrial dynamics and the potential molecular mechanism may be related to regulating p38 MAPK/PGC-1α pathway.

In this study, UHPLC-Q Exactive analysis was applied to identify the main components of BJD. The *in vitro* and *in vivo* model were conducted to investigate the improvement of BJD in preventing cancer-induced myotube atrophy. Also, we unveiled the molecular mechanism of BJD in regulating mitochondria dysfunction via the inhibition of p38 MAPK/PGC-1α signaling pathway. These results indicate that BJD plays an important role in cancer-induced myotube atrophy and could become a potential therapeutic drug for cancer cachexia treatment.

## Materials and Methods

### Cell Lines and Conditioned Medium

The lung adenocarcinoma cell lines Lewis cells and mouse C2C12 myoblast were purchased from Shanghai Institutes for Biological Sciences of Chinese Academy of Sciences (Shanghai, China) and routinely cultured in DMEM/high glucose medium (Invitrogen, United States) supplemented with 10% fetal bovine serum (Invitrogen, United States), 100 U/mL penicillin and 100 μg/mL streptomycin at 37°C in 5%CO_2_. The conditioned medium was collected as previously described (32). Briefly, Lewis cells were cultured in DMEM/high glucose medium at 37°C in 5%CO_2_ for 2 days. Then the culture medium was collected and filtered through a 0.22 μm membrane. The conditioned medium was conducted by mixed with fresh culture medium (DMEM/high glucose medium supplemented with 2% horse serum (Invitrogen, United States), 100 U/mL penicillin and 100 μg/mL streptomycin) in a ratio of 1:2, which named Lewis-cell conditioned medium (LCM).

### Preparation of the Extracts for BJD

Baoyuan Jiedu decoction was composed of six crude herbs: *Panax ginseng* C.A.Mey., *Aconitum carmichaelii* Debx., *Astragalus mongholicus* Bunge., *Angelica sinensis* (Oliv.) Diels., *Lonicera japonica* Thunb., and *Glycyrrhiza uralensis* Fisch. ex DC.in a ratio of 9:9:18:15:12:6 (9.0, 9.0, 18, 15, 12, and 6.0 g). The information on the drug materials were given in Supplementary Table 1. All the herbs were purchased from the Clinic Department of Zhejiang Chinese Medical University (Zhejiang, China) and identified by the Department of Pharmacy, Clinic Department of Zhejiang Chinese Medical University (Zhejiang, China). Baoyuan Jiedu decoction was prepared as hot-water extracts from the six crude herbs. Briefly, the mixture of *P. ginseng* C.A.Mey. and *A. carmichaelii* Debx. was macerated for 1 h and decocted for 1.5 h with 552 mL deionized water (1:8, w/v), *A. mongholicus* Bunge., *A. sinensis* (Oliv.) Diels., *L. japonica* Thunb., *G. uralensis* Fisch. ex DC., and 414 mL deionized water (1:6, w/v) was added and decocted for 1 h. The filtrates were blended and concentrated to 1.15 g crude drug/mL by rotary evaporation, which was stored at 4°C and filtered through a 0.22 μm membrane prior use.

### Liquid Chromatography and Mass Spectrometry

For UHPLC-Q Exactive analysis, the extracts obtained above was taken 200 μL, added 800 μL methanol and centrifuged at 20,000 rpm for 10 min at 4°C. The supernatant was filtered through a 0.22 μm membrane, then stored at 4°C. UHPLC-Q Exactive analysis was performed on a Thermo Scientific^TM^ Dionex^TM^ UltiMate^TM^ 3000 RSLC system equipped with a binary pump, autosampler, online vacuum degasser, and automatic thermostatic column oven, coupled with a Thermo Scientific^TM^ Q Exactive^TM^ MS (Thermo Fisher Scientific, Bremen, Germany) equipped with ESI. The data were recorded by Xcalibur 3.0 (Thermo Fisher Scientific). Chromatographic separation was performed using a Thermo Hypersil GOLD column (2.1 mm × 100 mm, 1.9 μm) at a flow rate of 0.4 mL/min. Sample (5 μL) was injected into the system, and the column temperature was maintained at 45°C. The mobile phase consisted of water containing 0.1% (v/v) formic acid (A) and acetonitrile (B). Linear gradient elution was applied (0–5 min, 5-30% B; 5–10 min, 30–40% B; 10–20 min, 40–65% B; 20–25 min, 65–90% B).

For MS detection, the operating parameters were as follows: HESI, spray voltage, 3.2 kV (Positive); capillary temp, 300°C; sheath gas pressure, 40 psi; auxiliary gas flow rate, 3 L/min; capillary temp, 300°C; scan mode: positive and negative ion switching scanning; scan range, m/z 100–1500, and the resolution (R) is 70,000.

### Reagents and Antibodies

Antibodies against Atrogin-1 (Cat# ab74023), MuRF-1 (Cat# ab172479), NRF-1 (Cat# ab175932), TFAM (Cat# ab131607), Cytochrome C (Cyt C) (Cat# ab13575), p38 MAPK (Cat# ab197348), p-p38 MAPK (Cat# ab195049), PGC-1α (Cat# ab54481), and GAPDH (Cat# ab9484) were purchased from Abcam (Cambridge, United States). Antibody against COXIV (Cat# bs1533) was purchased from Bioss (Beijing, China). p38 MAPK inhibitor SB203580(4-(4-Fluorophenyl)-2-(4-methylsulfinylphenyl)-5-(4-pyridyl)1H-imidazole) was purchased from Med Chem Express (NJ, United States). Megestrol acetate (Cat# B1377) was purchased from Ape × Bio (Houston, United States).

### *In vitro* Cancer-Induced Myotube Atrophy Model Construction

C2C12 cells were seeded in a 96-well plate and cultured in DMEM/high glucose medium. When the cell fusion reached 80–90%, the C2C12 cells were randomly divided into four groups: normal group (normal, *n* = 3), model group (model, *n* = 3), BJD group (BJD, *n* = 3), and p38 MAPK inhibitor group (SB203580, *n* = 3). The normal group was cultured in DMEM/high glucose medium without LCM, the model group was cultured in LCM. And the BJD group was cultured in LCM with 125 mg/mL BJD treatment, according to our previous study, BJD at concentrations up to 125 mg/mL has prevented C2C12 cells from LCM-induced myotube atrophy (32). Therefore, we chose a concentration of BJD at 125 mg/mL as an optimal dose in subsequent experiments. The p38 MAPK inhibitor group was cultured in LCM with 50 ng/mL SB203580 treatment. All the groups were continually cultured for 96 h. Morphological performance and transverse diameters of myotubes in C2C12 cells were observed every 24 h. The picture data were processed by Image J software (CA, United States).

### Mitochondrial Contents and ATPase Activity Assays

Mitochondrial contents in C2C12 cells were detected by 10-Ncnylacridine orange (NAO) stained. At the end of the experiment, the cells were washed with pre-cooled PBS and fixed with 0.2% glutaraldehyde at 4°C for 10 min. Then the NAO solution (10 μmol/L) was added in the dark for 10 min. The imaging was observed by a fluorescence microscope at 488 nm. The fluorescence intensity was proportional to the mitochondria contents. The level of mtDNA production was calculated by a TIANGEN Genomic DNA Kit (Cat# DP304, TIANGEN, China) and ATPase activity was detected by an ATP Colorimetric/Fluorometric Assay Kit (Cat# MAK190-1, Sigma, United States) according to the manufacturer’s instructions.

### *In vivo* Xenograft Cachexia Experiment

C26 tumor-bearing mice (20 ± 2) g and male BALB/c mice (20 ± 2) g were purchased from the Beijing Huakang Biotechnology Company (Beijing, China) and housed in an SPF (specific pathogen-free) and temperature-controlled (25 ± 2) °C environment with a 12 h light/dark cycle in Zhejiang Chinese Medical University Laboratory Animal Research Center (Hangzhou, China). The experiment began after mice adapted to the new environment for 1 week. The xenograft cachexia model was established as previously described (31). Briefly, C26 colorectal cancer cells were obtained from the C26 tumor-bearing mice after sacrificed and commercially countered to plate 2 × 10^6^/mL in flasks. And the BALB/c mice were injected 0.2 mL C26 cells subcutaneously through the right axillary skin. Then the mice were randomly divided equally (*n* = 10) into five groups: normal group (normal), model group (model), BJD group (BJD), megestrol acetate group (MA), and p38 MAPK inhibitor group (SB203580). In the BJD group, the dosage of BJD was 23 g/kg⋅d according to our previous study (30, 31). The dose of megestrol acetate was 24 mg/kg⋅d in the MA group, and the dose of SB203580 was 10 mg/kg in the p38 MAPK inhibitor group, while the normal and model group were given equal doses of normal saline. The MA group and SB203580 group were treated by intraperitoneal injection, while the normal group, model group, and BJD group were treated by gavage. The body weights were evaluated and recorded every day.

### Gastrocnemius Tissues Collection and H&E Strained

Mice were euthanized at the end of the experiment and the gastrocnemius tissues were collected for weighed and fixed in 4% phosphate-buffered paraformaldehyde, embedded in paraffin, and placed on microscope slides for routinely H&E histopathologic examination. The gastrocnemius index was calculated by the formula as the gastrocnemius weight (mg)/the body weight (g).

### Western Blotting

Tissues and cells were washed twice in 1 × PBS and lysed in radioimmunoprecipitation assay (RIPA) buffer on ice for 30 min. The lysate was centrifuged at 9000 rpm for 10 min at 4°C and the supernatant was collected. The protein concentration was quantified by a BCA Assay Kit (Thermo Fisher Scientific, United States) according to the manufacturer’s instructions. Samples were loaded and electrophoresed in 8–10% SDS-PAGE and transferred to PVDF membranes (Millipore, United States). Then the membranes were blocked in 5% non-fat milk powder diluted in TBS-T for 2 h before incubation with primary antibodies. Secondary antibodies included horseradish peroxidase (HRP)-conjugated goat anti-mouse IgG at 1:3000 dilution. The blots were visualized with enhanced chemiluminescence. The intensity of blots was analyzed by Image J. Each value was normalized by the respective value for GAPDH as an internal control.

### Real-Time PCR (qPCR) Analysis

Total RNAs were extracted from cells and gastrocnemius samples using Trizol reagent (Invitrogen, United States) according to the manufacturer’s instructions. Real-time PCR analyses were performed using SYBR Green (TOYOBO, Japan) and results were calculated based on the comparative cycle threshold method (2^–ΔΔCT^). β-actin was used to normalize the gene expression of other mRNAs. As for the analysis of the expression of mtDNA, β-globin was used as an internal gene. All of the primers were synthesized by Sangon Biotech (Shanghai, China). Primers sequences were as follows: Atrogin-1 forward 5′-GAGAACAGTATGGGGTCA-3′, reverse 5′-TAATAAAGTCTTGGGGTG-3′; MuRF-1 forward 5′-GCCACCTTCCTCTTGAGT-3′, reverse 5′-CCTTGTTCTGTC TTCCCC-3′; NRF-1 forward 5′-GCACAGAAGAGCAAAAG-3′, reverse 5′-CGAAAGCATACAGAAGG-3′; TFAM forward 5′-GTGGGGCGTGCTAAGAAC-3′, reverse 5′-GCTGACAGGC GAGGGTAT-3′; Cyt C forward 5′-ACCCTGATGGAGTATTTG-3′, reverse 5′-GCTATTAGTCTGCCCTTTC-3′; COXIV forward 5′-TGAGATGAACAAGGGCACCA-3′, reverse 5′-CA CCCAGTCACGATCAAAGG-3′; p38 MAPK forward 5′-GGT GTGTGCTGCTTTTGA-3′, reverse 5′-TGTAGGTCCTTTTGG CGT-3′; PGC-1α forward 5′-TTGGTGAAATTGAGGAATG-3′, reverse 5′-CACAGGTGTAACGGTAGGT-3′; ATP synthase-F subunit β peptide forward 5′-TCTTGTGGGGCGTGTGG-3′, reverse 5′-GCGGGAGCGGTTCGTAG-3′; β-actin forward 5′-TCAGCAATGCCTGGGTACAT-3′, reverse 5′-ATCACTATT GGCAACGAGCG-3′; β-globin forward 5′-TGATGCTGAGAA GGCTGCT-3′, reverse 5′-CCTCTGGGTCCAAGGGTAG-3′.

### Statistical Analysis

Statistical analyses were performed using SPSS 26.0 (SPSS Inc., United States) and data were presented as means ± SD. Differences between multiple comparisons were assessed using one-way analysis of variance (ANOVA) with Tukey’s multiple comparison test whereas two groups were analyzed using an unpaired Student’s *t-*test. *P* < 0.05 was considered to be statistically significant.

## Results

### Identification of the Main Components in the BJD Extract

**FIGURE 1:**
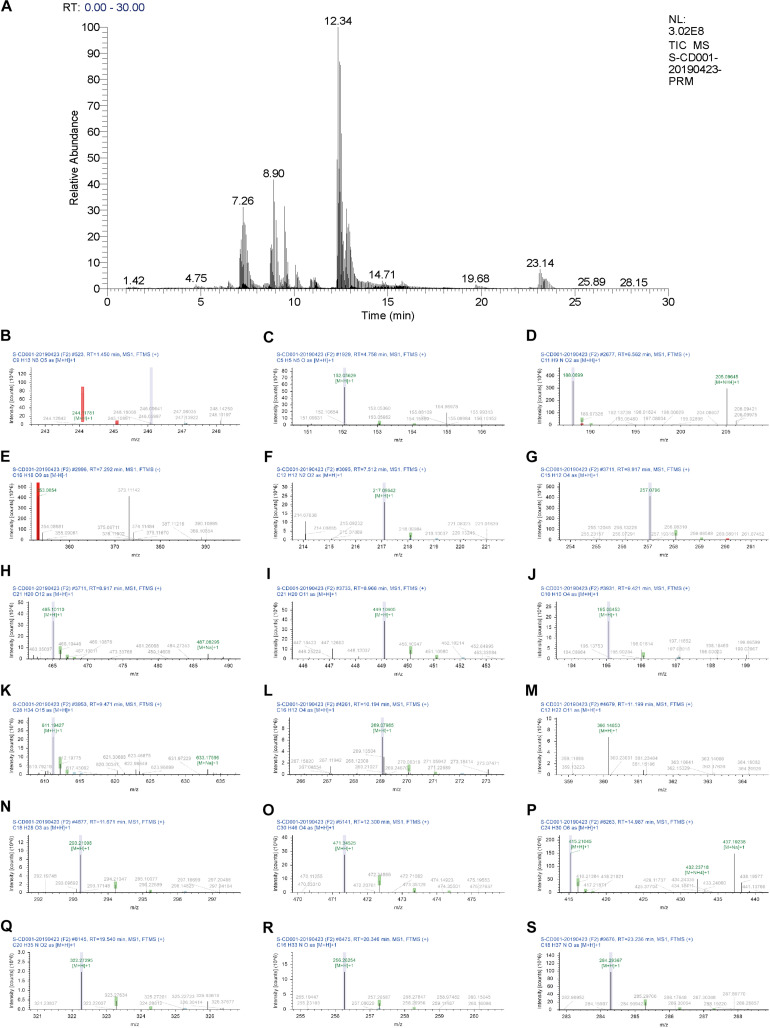
UHPLC chromatogram profiles of BJD. **(A)** Total ion current chromatograms of BJD. Secondary mass spectrum of the 18 main components of BJD: **(B)** Cytidine. **(C)** Guanine. **(D)** Indole-3-acrylic acid. **(E)** Chlorogenic acid. **(F)** 2,3,4,9-Tetrahydro-1H-β-carboline-3-carboxylic acid. **(G)** Isoliquiritigenin. **(H)** Quercetin-3β-D-glucoside. **(I)** Cynaroside. **(J)** Ferulic acid. **(K)** Hesperidin. **(L)** Formononetin. **(M)**α-Lactose. **(N)** 9S,13R-12-Oxophytodienoic acid. **(O)** 18-β-Glycyrrhetinic acid. **(P)** Bis(4-ethylbenzylidene)sorbitol. **(Q)** α-Linolenoyl ethanolamide. **(R)** Hexadecanamide. **(S)** Stearamide.

Eighteen chemical components of BJD were identified by UHPLC-Q Exactive analysis. Besides, some chemical markers, such as chlorogenic acid, ferulic acid, were used for quality control of BJD. The total ion current diagram and the secondary mass spectrum results of the chemical components were shown in Table 1 and Figure 1.

**TABLE 1:** Main components identified in BJD.

No.	Name	Formula	RT (min)	Molecular weight
1	Cytidine	C_9_H_13_N_3_O_5_	1.461	244.1284
2	Guanine	C_5_H_5_N_5_O	4.763	152.0562
3	Indole-3-acrylic acid	C_11_H_9_NO_2_	6.556	188.0699
4	Chlorogenic acid	C_16_H_18_O_9_	7.309	353.0854
5	2,3,4,9-Tetrahydro-1H-β-carboline-3-carboxylic acid	C_12_H_12_N_2_O_2_	7.497	217.0964
6	Isoliquiritigenin	C_15_H_12_O_4_	8.939	257.0796
7	Quercetin-3β-D-glucoside	C_21_H_20_O_12_	8.939	465.1011
8	Cynaroside	C_21_H_20_O_11_	8.946	449.1061
9	Ferulic acid	C_10_H_10_O_4_	9.41	195.0645
10	Hesperidin	C_28_H_34_O_15_	9.449	611.1962
11	Formononetin	C_16_H_12_O_4_	10.189	267.0796
12	α-Lactose	C_12_H_22_O_11_	11.184	360.1485
13	9S,13R-12-Oxophytodienoic acid	C_18_H_28_O_3_	11.645	293.2101
14	18-β-Glycyrrhetinic acid	C_30_H_46_O_4_	12.324	471.3452
15	Bis(4-ethylbenzylidene)sorbitol	C_24_H_30_O_6_	14.997	415.2104
16	α-Linolenoyl ethanolamide	C_20_H_35_NO_2_	19.533	322.2729
17	Hexadecanamide	C_16_H_33_NO	20.366	256.2625
18	Stearamide	C_18_H_37_NO	23.246	284.2936

### BJD Ameliorated LCM-Induced C2C12 Myotube Atrophy

**FIGURE 2:**
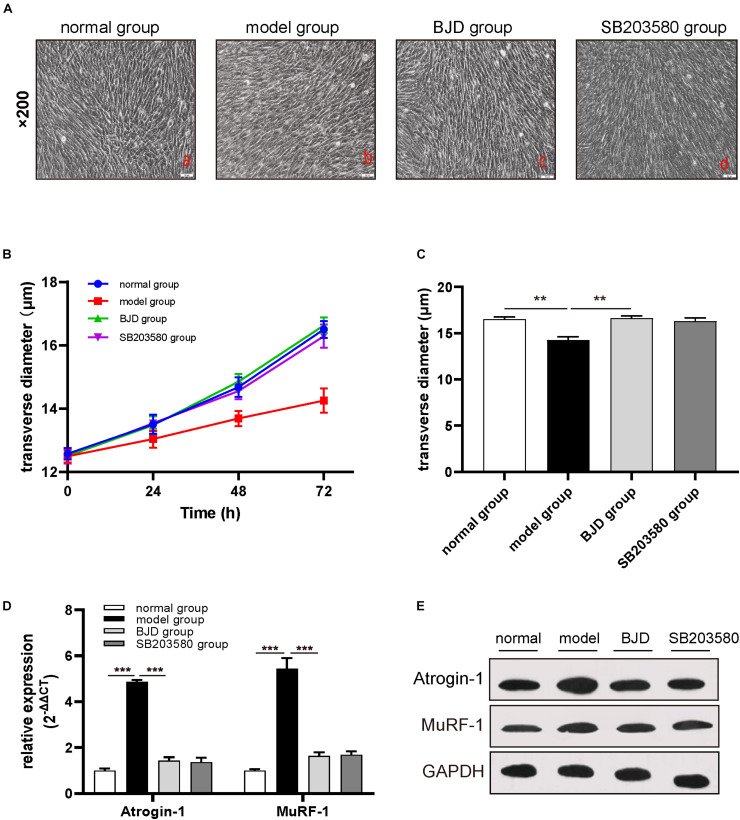
BJD prevented LCM-induced myotube atrophy in C2C12 cells. **(A)** The morphological changes of myotubes in C2C12 cells (×200 magnification; scale bar: 50 μm). **(B,C)** The transverse diameters of myotubes in C2C12 cells (μm, *n* = 3). **(D)** The relative expression of Atrogin-1 and MuRF-1 was detected by RT-qPCR, with β-actin was used as an internal gene. **(E)** The expression of Atrogin-1 and MuRF-1 was detected by Western blotting, GAPDH was used as a loading control. The data are presented as the mean ± SD. **P < 0.01, ***P < 0.001.

The C2C12 myoblast is a classical cell for studying cancer-induced myotube atrophy. To examine whether BJD could prevent myotubes atrophy, C2C12 cells were induced by LCM and incubated with BJD for 72 h. As a result, LCM postponed the differentiation of myotubes in C2C12 cells while BJD promoted the differentiation of myotubes as compared with the normal group (Figure 2A). Measurements of the myotube transverse diameter showed that BJD treatment and SB203580 significantly increased the myotube thickness compared with the model group (Figures 2B,C).

### BJD Decreased the Expression of Atrogin-1 and MuRF-1 in C2C12 Cells

The high expressions of muscle-specific atrophy marker proteins, such as Atrogin-1 and MuRF-1, caused the degradation of myotubes. Therefore, to further investigate whether the effect of BJD on improving the myotubes atrophy was achieved through modulating these proteins expressions, we used Western blotting and qPCR to determine their protein and mRNA levels. BJD treatment significantly decreased the expressions of Atrogin-1 and MuRF-1 both in protein and mRNA compared with the model group as predicted (Figures 2D,E). Besides, SB203580 had a similar impact with BJD but not significant.

### BJD Promoted the Generation of Mitochondria

**FIGURE 3:**
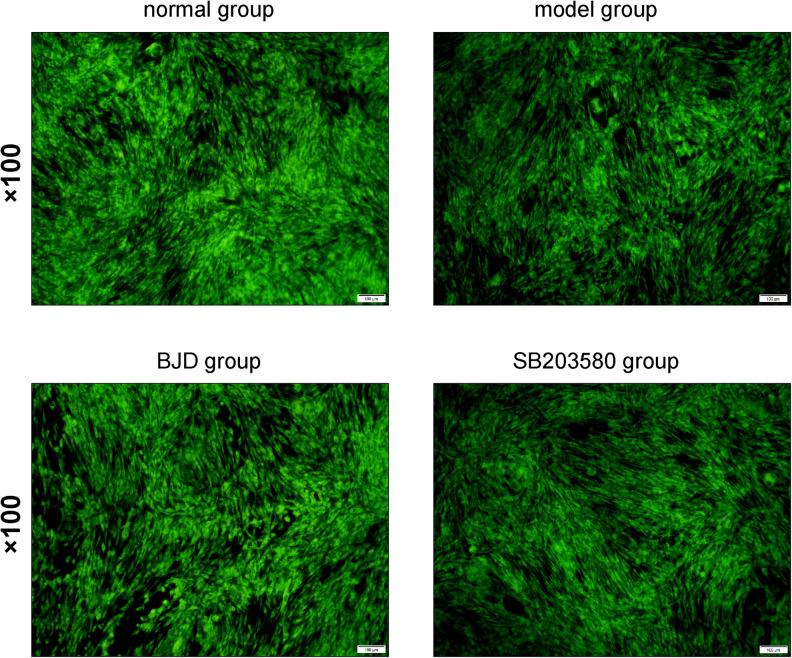
BJD enhanced the mitochondria generation. The C2C12 cells were washed with pre-cooled PBS and fixed with 0.2% glutaraldehyde at 4°C for 10 min. Then the NAO solution (10 μmol/L) was added in the dark for 10 min. The imaging was observed by a fluorescence microscope at 488 nm. The picture was ×100 magnification, and the scale bar is 100 μm.

**FIGURE 4:**
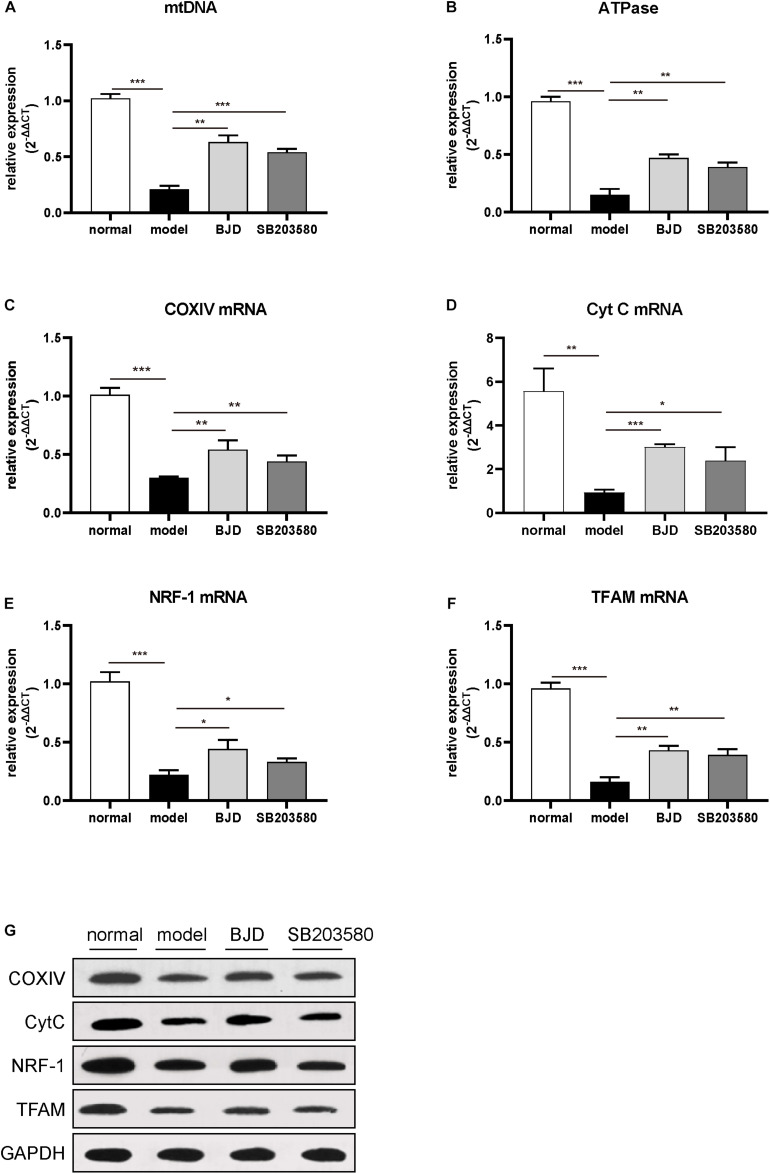
BJD improved mtDNA and increased the expression of ATPase, NRF-1, TFAM, COXIV, and CytC. **(A)** The relative expression of mtDNA, with β-globin was used as an internal gene. **(B–F)** The relative expression of ATPase, NRF-1, TFAM, COXIV, and CytC, with β-actin was used as an internal gene. **(G)** The expression of NRF-1, TFAM, COXIV, and CytC was detected by Western blotting, GAPDH was used as a loading control. The data are presented as the mean ± SD. *P < 0.05, **P < 0.01, ***P < 0.001.

Mitochondria are almost enriched skeletal muscle and responsible for the metabolic function and physiological or pathological responses. Therefore, to investigate the effect of BJD on the generation of mitochondria, C2C12 cells were collected and mitochondrial contents were detected by NAO staining. As a result, BJD treatment increased the fluorescence intensity, which is similar with the result of SB203580, whereas fluorescence intensity was slight in the model group (Figure 3). Mitochondrial DNA is an important indicator for mitochondrial biosynthesis, therefore, to determine whether BJD could increase mtDNA synthesis, we detected the expression of mtDNA by qPCR. As a result, BJD increased the expression of mtDNA compared with the model group, as similar in SB203580 treatment (Figure 4A).

### BJD Increased the Expression of NRF-1, TFAM, COXIV, Cyt C

Nuclear respiratory factor and TFAM are two essential mediators in promoting mtDNA biosynthesis (33). The results of WB and qPCR showed that BJD significantly increased the expressions of NRF-1 and TFAM compared with the model group (Figures 4B,C). Treatment with BJD also enhanced mitochondrial oxidative phosphorylation, which increased the expression of ATPase (Figure 4D), COXIV, and Cyt C (Figures 4E–G), compared with the model group.

### BJD Inhibited p38 MAPK/PGC-1α Signaling Pathway

**FIGURE 5:**
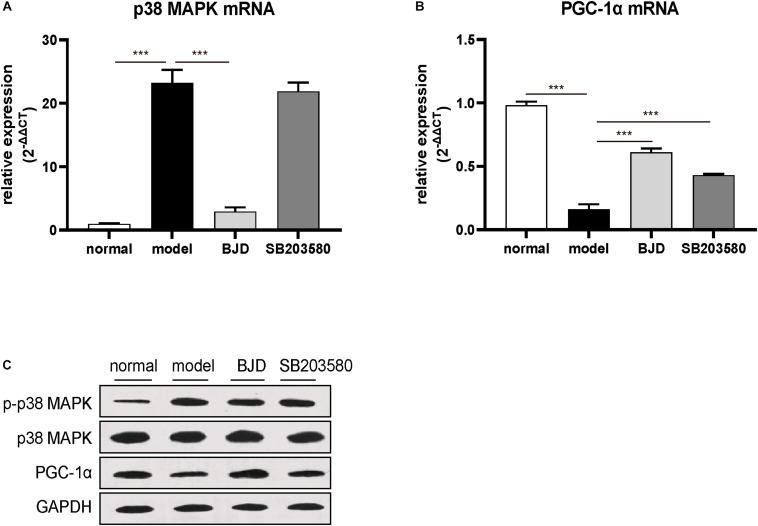
BJD treatment inhibited p38 MAPK/PGC-1α signaling pathway. **(A,B)** The relative expression of p38 MAPK and PGC-1, with β-actin was used as an internal gene. **(C)** The expression of p-p38 MAPK, p38 MAPK, and PGC-1α was detected by Western blotting, GAPDH was used as a loading control. The data are presented as the mean ± SD. ***P < 0.001.

The p38 MAPK/PGC-1α signaling pathway regulates the mitochondrial biosynthesis and energy metabolism and causes muscular atrophy. Therefore, to detect whether BJD could regulate p38 MAPK/PGC-1α signaling pathway to enhance mitochondrial function, their protein and mRNA levels were measured by Western blot and qPCR, respectively. Treatment with BJD significantly decreased the expression of p38 MAPK and p38 MAPK phosphorylation (Figures 5A,C) whereas increased the expression of PGC-1α (Figures 5B,C), compared with the model group. Similar to BJD, SB203580 treatment also promoted the expression of PGC-1α, but did not affect the expression of p38 MAPK.

### BJD Alleviated the Gastrocnemius Atrophy in Xenograft Cachexia Mice

**FIGURE 6:**
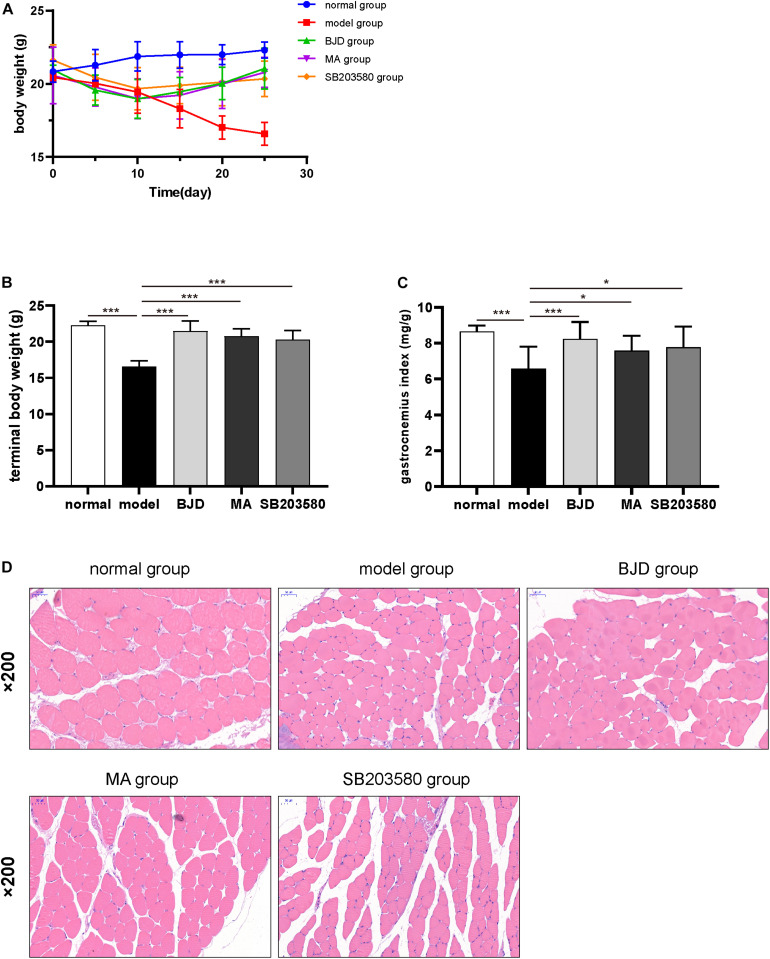
BJD alleviated gastrocnemius atrophy in xenograft cachexia mice. **(A)** The changes in body weight in each group. **(B)** The terminal body weights. **(C)** The gastrocnemius index. **(D)** The pathological pictures of gastrocnemius muscle stained by H&E (×200 magnification; scale bar: 50 μm). The data are presented as the mean ± SD. *P < 0.05, ***P < 0.001.

**FIGURE 7:**
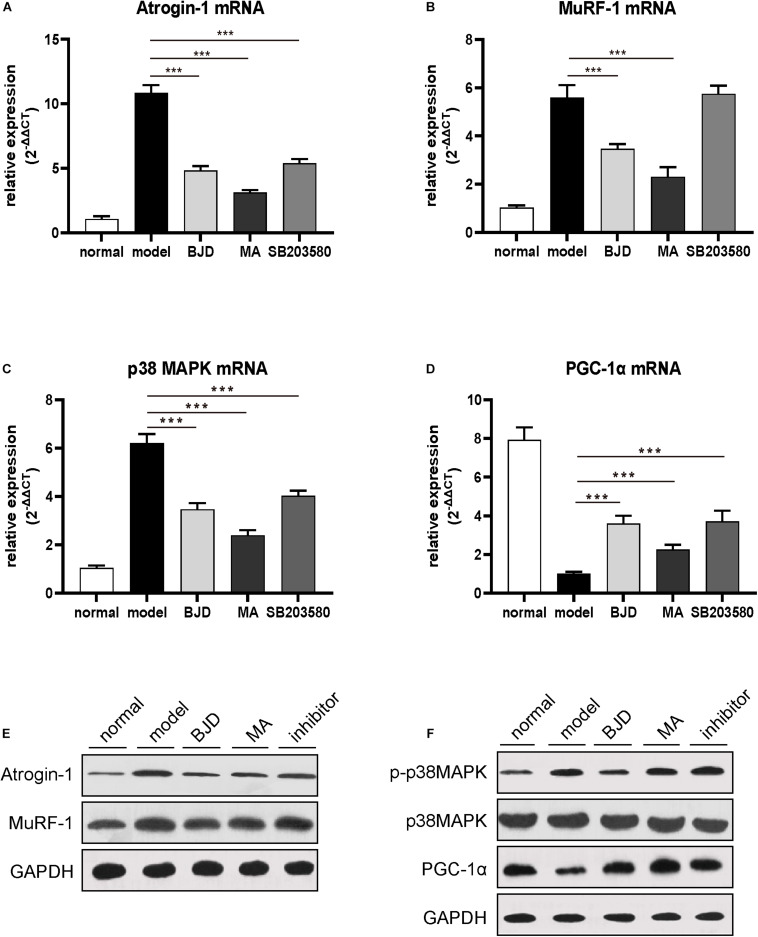
BJD inhibited p38 MAPK/PGC-1α singling pathway in mice. **(A–D)** The relative expression of Atrogin-1, MuRF-1, p38 MAPK, and PGC-1α was detected by RT-qPCR, with β-actin was used as an internal gene. **(E–F)** The expression of Atrogin-1, MuRF-1, p-p38 MAPK, p38 MAPK, and PGC-1α was detected by Western blotting, GAPDH was used as a loading control. The data are presented as the mean ± SD. ***P < 0.001.

To verify the effect of BJD on cancer cachexia model *in vivo*, mice were treated with or without BJD. And the results showed that the ongoing body weight loss was prevented by BJD treatment (Figures 6A,B). Similarly, megestrol acetate and SB203580 also exhibited protection on body weight loss. Moreover, BJD treatment improved the gastrocnemius index compared with the model group (Figure 6C), the same as the megestrol acetate and SB203580 treatment. Additionally, BJD treatment improved the diameters of the gastrocnemius muscle, whereas megestrol acetate and SB203580 treatment had a similar effect (Figure 6D). Furthermore, BJD treatment significantly decreased the expression of Atrogin-1 and MuRF-1 protein and mRNA levels, and megestrol acetate had a similar effect (Figures 7A,B,E). Besides, BJD treatment inhibited the expression of p38 MAPK and p38 MAPK phosphorylation whereas increased the expression of PGC-1α (Figures 7C,D,F). Interestingly, SB203580 did not affect MuRF-1 expression but slightly decreased the expression of p38 MAPK (Figures 7B,E,F).

## Discussion

Cancer cachexia, considered as a multifactorial syndrome, frequently occurs in advanced cancer patients (1, 4, 34). The loss of body weight, especially the loss of whole-body skeletal muscle mass with or without fat consumption, is the most prominent clinical characteristic of cachexia (35, 36). The progressive weight loss not only causes the intolerance of patient to radiotherapy and chemotherapy but also has extensive impact on the quality of life (37). However, the therapeutic approaches or agents for cancer cachexia are currently limited.

Traditional Chinese Medicine is widely used in relieving cachexia symptoms (38, 39). According to the theory of TCM, cancer cachexia belongs to a kind of consumptive disease, and the Yang-qi deficiency along with toxin stagnating is considered as its main pathogenesis. BJD, a Chinese formula consisted of 6 herbals including *P. ginseng* C.A.Mey., *A. carmichaelii* Debx., *A. mongholicus* Bunge., *A. sinensis* (Oliv.) Diels., *L. japonica* Thunb., and *G. uralensis* Fisch. ex DC., has the function of benefiting Yang-qi, resolving toxin and strengthening muscles. In this study, we identified the main components of BJD through UHPLC-Q Exactive analysis and some chemical markers that provided the basis for the pharmacology. Some of these components, such as luteolin, ferulic acid, have anti-inflammatory, immunomodulatory, and anti-tumor effects (40, 41). Chlorogenic acid and ferulic acid showed obvious anti-cancer effect (42, 43). Notably, ginsenoside Rb1 is reported to contribute to recover the cancer cachexia state (44). Ginsenoside Rb3, the main constituent of ginseng, has the function of upregulating myotube formation and mitochondrial function (45). Therefore, these results provide a material basis of BJD for cachexia treatment.

The degradation of muscle-related proteins induced by systemic inflammation, UPP, and mitochondrial dysfunction, is closely associated with skeletal muscle atrophy in cachexia (46). TNF-α, a strong inflammatory cytokine, can activate the NF-κB-mediated transcription of MuRF-1, thereby leading to increased protein degradation (8, 9). Besides, the NF-κB signaling pathway inhibits the expression of mitochondrial genes so that reduces mitochondrial biosynthesis and reduces its oxidative phosphorylation capacity and ATP production (22, 47). Therefore, activated cytokines can up-regulate the expression of E3 ubiquitin ligase by activating UPP and accelerate muscle protein degradation (46). Previously, our study revealed that BJD prevented the loss of boy weight and induced C2C12 myotube differentiation. BJD also reduced TNF-α level and inhibited the expression of MuRF-1 by inhibiting the UPP-mediated muscle protein degradation (30–32). In this study, we used a xenograft cachexia mice model and found that BJD prevented body weight loss and improved the pathological process of muscle atrophy. These results indicate that BJD has a potential therapeutic effect on cancer cachexia.

Mitochondria play important roles in maintaining the generation and function of skeletal muscle. Recently, it has been reported that the capability of mitochondria generation, oxidative phosphorylation function, and ATP synthesis is impaired in cancer-induced cachexia (1, 17, 18, 48). Therefore, improving mitochondria function may become a new therapeutic target in cancer cachexia (49). We found that BJD can promote C2C12 cells differentiation, and the transverse diameter of the myotube gradually increases along with the intervention time. Besides, after BJD treatment, the mitochondrial fluorescence intensity and the expression of mtDNA increased significantly in C2C12 cells. As NRF-1 and TFAM are key factors in mitochondrial biosynthesis, BJD treatment can increase their expression levels, and COXIV and Cty C, which are two major oxidative phosphorylation markers, were up-regulated by BJD treatment. These results indicate that BJD can improve mitochondrial biosynthesis and increase oxidative phosphorylation function.

The p38 MAPK/PGC-1α signaling pathway mainly regulates mitochondrial functions from several aspects, including biosynthesis and oxidative phosphorylation. In the cancer cachexia condition, inflammatory factors, such as TNF-α, IL-1, and IL-6, can activate p38 MAPK signaling, thereby inhibiting PGC-1α transcription and reducing energy production (22, 50, 51). Proliferator-activated receptor-gamma coactivator 1, a key factor for mitochondrial synthesis, mainly distributes in skeletal muscle, heart, and other tissues that require high energy (33). Emerging evidence has reported that PGC-1α activation induces the expression of mitochondrial biogenesis factors, such as mtDNA, NRF-1, TFAM, and Cyt C (20, 50). Mice that knocked out PGC-1α showed a decreased mitochondrial capacity in skeletal muscle. On the contrary, increasing the expression of PGC-1α can promote the expression of mitochondrial protein and restore mitochondrial function (52). We found that BJD significantly decreased the expression of p38 MAPK and its phosphorylation, and increased the expression of PGC-1α both *in vitro* and *in vivo* experiments. Besides, we found that SB203580 did not inhibit the expression of p38 MAPK in C2C12 cells, but slightly up-regulated PGC-1α. The reason is that SB203580 can inhibit the activity of p38 kinase on its substrates including p38 itself and its downstream substrate. Proliferator-activated receptor-gamma coactivator 1, as one of the downstream substrates of p38 MAPK, was showed up-regulation of protein and mRNA expression after SB203580 treatment, which indicated that SB203580 inhibited the process of p38 kinase on PGC-1α activity. This result is consistent with the previous report (53). However, SB203580 does not affect the upstream activation events of p38 MAPK, such as TNF-α or IL-6 induced p38 MPAK activation progress (54, 55). Therefore, the expression of p38 MAPK in the SB203580 group showed no significant difference compared with the model group. This may be due to the activation of p38 MAPK by upstream events. Our previous studies reveal that BJD can reduce the content of TNF-α and IL-6 (32). Therefore, it explains the phenomenon that BJD reduced the expression of p38 MAPK while SB203580 did not affect its expression *in vitro*.

In conclusion, the present findings reveal that BJD can improve mitochondrial function by regulating the p38 MAPK/PGC-1α signaling pathway, thereby preventing the cancer-induced myotube atrophy. Although our data confirm that BJD may be a potential drug for the treatment of cancer cachexia, there are some necessary investigations, such as conducting the toxicology experiments, screening and verifying effective small molecules of BJD for cachexia treatment, which are desirable for future work.

## Data Availability Statement

All datasets generated for this study are included in the article/Supplementary Material.

## Ethics Statement

The studies involving animal study were reviewed and approved by Animal Ethical and Welfare Committee of ZCMU, Zhejiang Chinese Medical University.

## Author Contributions

XJ, HZ, and DW contributed to the study design, data analysis, and manuscript drafting. DW, XZ, and WC contributed to the animal experiment. QB and WC carried out the *in vitro* experiment. QB, JR, XY, and JW contributed to the data acquisition and analysis. All authors contributed to the article and approved the submitted version.

## Conflict of Interest

The authors declare that the research was conducted in the absence of any commercial or financial relationships that could be construed as a potential conflict of interest.

## References

[1] Fearon KC Glass DJ Guttridge DC. Cancer cachexia: mediators, signaling, and metabolic pathways. *Cell Metab.* (2012) 16:153–66. 10.1016/j.cmet.2012.06.011 22795476

[2] Feng X Wang Z Wang F Lu T Xu J Ma X Dual function of VGLL4 in muscle regeneration. *EMBO J.* (2019) 38:e101051. 10.15252/embj.2018101051 31328806 PMC6717915

[3] Fearon K Strasser F Anker SD Bosaeus I Bruera E Fainsinger RL Definition and classification of cancer cachexia: an international consensus. *Lancet Oncol.* (2011) 12:489–95. 10.1016/s1470-2045(10)70218-7 21296615

[4] Turner DC Kondic AG Anderson KM Robinson AG Garon EB Riess JW Pembrolizumab exposure-response assessments challenged by association of cancer cachexia and catabolic clearance. *Clin Cancer Res.* (2018) 24:5841–9. 10.1158/1078-0432.Ccr-18-0415 29891725

[5] Schwartsburd P. Cancer-induced reprogramming of host glucose metabolism: “Vicious Cycle” supporting cancer progression. *Front Oncol.* (2019) 9:218. 10.3389/fonc.2019.00218 31019893 PMC6458235

[6] Counts BR Fix DK Hetzler KL Carson JA. The effect of estradiol administration on muscle mass loss and cachexia progression in female Apc (Min/+) mice. *Front Endocrinol.* (2019) 10:720. 10.3389/fendo.2019.00720 31736871 PMC6838005

[7] Dolan RD Almasaudi AS Dieu LB Horgan PG McSorley ST McMillan DC. The relationship between computed tomography-derived body composition, systemic inflammatory response, and survival in patients undergoing surgery for colorectal cancer. *J Cachexia Sarcopenia Muscle.* (2019) 10:111–22. 10.1002/jcsm.12357 30460764 PMC6438413

[8] Zhang G Liu Z Ding H Miao H Garcia JM Li YP. Toll-like receptor 4 mediates Lewis lung carcinoma-induced muscle wasting via coordinate activation of protein degradation pathways. *Sci Rep.* (2017) 7:2273. 10.1038/s41598-017-02347-2 28536426 PMC5442131

[9] Cai D Frantz JD Tawa NE Jr. Melendez PA Oh BC Lidov HG IKKbeta/NF-kappaB activation causes severe muscle wasting in mice. *Cell.* (2004) 119:285–98. 10.1016/j.cell.2004.09.027 15479644

[10] Nicolini A Ferrari P Masoni MC Fini M Pagani S Giampietro O Malnutrition, anorexia and cachexia in cancer patients: a mini-review on pathogenesis and treatment. *Biomed Pharmacother.* (2013) 67:807–17. 10.1016/j.biopha.2013.08.005 24035652

[11] Gomes MD Lecker SH Jagoe RT Navon A Goldberg AL. Atrogin-1, a muscle-specific F-box protein highly expressed during muscle atrophy. *Proc Natl Acad Sci USA.* (2001) 98:14440–5. 10.1073/pnas.251541198 11717410 PMC64700

[12] Halle JL Pena GS Paez HG Castro AJ Rossiter HB Visavadiya NP Tissue-specific dysregulation of mitochondrial respiratory capacity and coupling control in colon-26 tumor-induced cachexia. *Am J Physiol Regul Integr Comp Physiol.* (2019) 317:R68–82. 10.1152/ajpregu.00028.2019 31017805

[13] Collins P Bing C McCulloch P Williams G. Muscle UCP-3 mRNA levels are elevated in weight loss associated with gastrointestinal adenocarcinoma in humans. *Br J Cancer.* (2002) 86:372–5. 10.1038/sj.bjc.6600074 11875702 PMC2375209

[14] Favaro G Romanello V Varanita T Andrea Desbats M Morbidoni V Tezze C DRP1-mediated mitochondrial shape controls calcium homeostasis and muscle mass. *Nat Commun.* (2019) 10:2576. 10.1038/s41467-019-10226-9 31189900 PMC6561930

[15] Ren Y Li Y Lv J Guo X Zhang J Zhou D Parthenolide regulates oxidative stress-induced mitophagy and suppresses apoptosis through p53 signaling pathway in C2C12 myoblasts. *J Cell Biochem.* (2019) 120:15695–708. 10.1002/jcb.28839 31144365

[16] Constantinou C Fontes de Oliveira CC Mintzopoulos D Busquets S He J Kesarwani M Nuclear magnetic resonance in conjunction with functional genomics suggests mitochondrial dysfunction in a murine model of cancer cachexia. *Int J Mol Med.* (2011) 27:15–24. 10.3892/ijmm.2010.557 21069263 PMC3712618

[17] Hardee JP Counts BR Gao S VanderVeen BN Fix DK Koh HJ Inflammatory signalling regulates eccentric contraction-induced protein synthesis in cachectic skeletal muscle. *J Cachexia Sarcopenia Muscle.* (2018) 9:369–83. 10.1002/jcsm.12271 29215198 PMC5879978

[18] White JP Baltgalvis KA Puppa MJ Sato S Baynes JW Carson JA. Muscle oxidative capacity during IL-6-dependent cancer cachexia. *Am J Physiol Regul Integr Comp Physiol.* (2011) 300:R201–11. 10.1152/ajpregu.00300.2010 21148472 PMC3043802

[19] Bing C Brown M King P Collins P Tisdale MJ Williams G. Increased gene expression of brown fat uncoupling protein (UCP)1 and skeletal muscle UCP2 and UCP3 in MAC16-induced cancer cachexia. *Cancer Res.* (2000) 60:2405–10. 10811117

[20] Wu Z Puigserver P Andersson U Zhang C Adelmant G Mootha V Mechanisms controlling mitochondrial biogenesis and respiration through the thermogenic coactivator PGC-1. *Cell.* (1999) 98:115–24. 10.1016/s0092-8674(00)80611-x 10412986

[21] Jäger S Handschin C St-Pierre J Spiegelman BM. AMP-activated protein kinase (AMPK) action in skeletal muscle via direct phosphorylation of PGC-1alpha. *Proc Natl Acad Sci USA.* (2007) 104:12017–22. 10.1073/pnas.0705070104 17609368 PMC1924552

[22] Puigserver P Rhee J Lin J Wu Z Yoon JC Zhang CY Cytokine stimulation of energy expenditure through p38 MAP kinase activation of PPARgamma coactivator-1. *Mol Cell.* (2001) 8:971–82. 10.1016/s1097-2765(01)00390-2 11741533

[23] Knutti D Kressler D Kralli A. Regulation of the transcriptional coactivator PGC-1 via MAPK-sensitive interaction with a repressor. *Proc Natl Acad Sci USA.* (2001) 98:9713–8. 10.1073/pnas.171184698 11481440 PMC55518

[24] Fan M Rhee J St-Pierre J Handschin C Puigserver P Lin J Suppression of mitochondrial respiration through recruitment of p160 myb binding protein to PGC-1alpha: modulation by p38 MAPK. *Genes Dev.* (2004) 18:278–89. 10.1101/gad.1152204 14744933 PMC338281

[25] Zetser A Gredinger E Bengal E. p38 mitogen-activated protein kinase pathway promotes skeletal muscle differentiation. Participation of the Mef2c transcription factor. *J Biol Chem.* (1999) 274:5193–200. 10.1074/jbc.274.8.5193 9988769

[26] Kim J Won KJ Lee HM Hwang BY Bae YM Choi WS p38 MAPK participates in muscle-specific ring finger 1-mediated atrophy in cast-immobilized rat gastrocnemius muscle. *Korean J Physiol Pharmacol.* (2009) 13:491–6. 10.4196/kjpp.2009.13.6.491 20054497 PMC2802311

[27] Zhang X Jing S Lin H Sun W Jiang W Yu C Anti-fatigue effect of anwulignan via the NRF2 and PGC-1α signaling pathway in mice. *Food Funct.* (2019) 10:7755–66. 10.1039/c9fo01182j 31696200

[28] Sun X Xu X Chen Y Guan R Cheng T Wang Y Danggui buxue decoction sensitizes the response of non-small-cell lung cancer to gemcitabine via regulating deoxycytidine kinase and p-glycoprotein. *Molecules.* (2019) 24:2011. 10.3390/molecules24102011 31130654 PMC6572355

[29] Peng W Zhang S Zhang Z Xu P Mao D Huang S Jianpi Jiedu decoction, a traditional Chinese medicine formula, inhibits tumorigenesis, metastasis, and angiogenesis through the mTOR/HIF-1α/VEGF pathway. *J Ethnopharmacol.* (2018) 224:140–8. 10.1016/j.jep.2018.05.039 29852266

[30] Zong X Zhang Y Zhang H Ji X. Mechanism of baoyuan jiedu decoction in alleviating muscle atrophy in Apcmin/+ cachexia mice. *Chin J Exp Trad Med Formul.* (2019) 25:19–24.

[31] Zhang Y Han X Ouyang B Wu Z Yu H Wang Y Chinese herbal medicine baoyuan jiedu decoction inhibited muscle atrophy of cancer cachexia through Atrogin-l and MuRF-1. *Evid Based Complement Alternat Med.* (2017) 2017:6268378. 10.1155/2017/6268378 28286533 PMC5329682

[32] Zhang H Zong X Deng T ZHao R Ji X. Mechanisms of Baoyuan Jiedu decoction in the intervention of carcinogenic muscular atrophy through inhibiting cytokins-ubiquitin-proteasome pathway. *J Beijing Univ Trad Chin Med.* (2018) 41:642–7.

[33] Arany Z. PGC-1 coactivators and skeletal muscle adaptations in health and disease. *Curr Opin Genet Dev.* (2008) 18:426–34. 10.1016/j.gde.2008.07.018 18782618 PMC2629557

[34] Argilés JM Busquets S Stemmler B López-Soriano FJ. Cancer cachexia: understanding the molecular basis. *Nat Rev Cancer.* (2014) 14:754–62. 10.1038/nrc3829 25291291

[35] Acharyya S Ladner KJ Nelsen LL Damrauer J Reiser PJ Swoap S Cancer cachexia is regulated by selective targeting of skeletal muscle gene products. *J Clin Invest.* (2004) 114:370–8. 10.1172/jci20174 15286803 PMC484974

[36] Baltgalvis KA Berger FG Peña MM Davis JM White JP Carson JA. Muscle wasting and interleukin-6-induced atrogin-I expression in the cachectic Apc. (Min/+) mouse. *Pflugers Arch.* (2009) 457:989–1001. 10.1007/s00424-008-0574-6 18712412 PMC2867110

[37] Fearon K Arends J Baracos V. Understanding the mechanisms and treatment options in cancer cachexia. *Nat Rev Clin Oncol.* (2013) 10:90–9. 10.1038/nrclinonc.2012.209 23207794

[38] Wu TH Yeh KY Wang CH Wang H Li TL Chan YL The combination of *Astragalus membranaceus* and *Angelica sinensis* inhibits lung cancer and cachexia through its immunomodulatory function. *J Oncol.* (2019) 2019:9206951. 10.1155/2019/9206951 31781219 PMC6875282

[39] Kang HJ Jeong MK Park SJ Jun HJ Yoo HS. Efficacy and safety of Yukgunja-Tang for treating anorexia in patients with cancer: the protocol for a pilot, randomized, controlled trial. *Medicine.* (2019) 98:e16950. 10.1097/md.0000000000016950 31577697 PMC6783206

[40] Jiang ZQ Li MH Qin YM Jiang HY Zhang X Wu MH. Luteolin inhibits tumorigenesis and induces apoptosis of non-small cell lung cancer cells via regulation of MicroRNA-34a-5p. *Int J Mol Sci.* (2018) 19:447. 10.3390/ijms19020447 29393891 PMC5855669

[41] Zhang X Lin D Jiang R Li H Wan J Li H. Ferulic acid exerts antitumor activity and inhibits metastasis in breast cancer cells by regulating epithelial to mesenchymal transition. *Oncol Rep.* (2016) 36:271–8. 10.3892/or.2016.4804 27177074

[42] Huang S Wang LL Xue NN Li C Guo HH Ren TK Chlorogenic acid effectively treats cancers through induction of cancer cell differentiation. *Theranostics.* (2019) 9:6745–63. 10.7150/thno.34674 31660066 PMC6815948

[43] Das U Manna K Adhikary A Mishra S Saha KD Sharma RD Ferulic acid enhances the radiation sensitivity of lung and liver carcinoma cells by collapsing redox homeostasis: mechanistic involvement of Akt/p38 MAPK signalling pathway. *Free Radic Res.* (2019) 53:944–67. 10.1080/10715762.2019.1655559 31576765

[44] Lu S Zhang Y Li H Zhang J Ci Y Han M. Ginsenoside Rb1 can ameliorate the key inflammatory cytokines TNF-α and IL-6 in a cancer cachexia mouse model. *BMC Complement Med Ther.* (2020) 20:11. 10.1186/s12906-019-2797-9 32020864 PMC7076885

[45] Lee SJ Bae JH Lee H Lee H Park J Kang JS Ginsenoside Rg3 upregulates myotube formation and mitochondrial function, thereby protecting myotube atrophy induced by tumor necrosis factor-alpha. *J Ethnopharmacol.* (2019) 242:112054. 10.1016/j.jep.2019.112054 31271820

[46] Tisdale MJ. Loss of skeletal muscle in cancer: biochemical mechanisms. *Front Biosci.* (2001) 6:D164–74. 10.2741/tisdale 11171557

[47] Johnson RF Witzel II Perkins ND. p53-dependent regulation of mitochondrial energy production by the RelA subunit of NF-κB. *Cancer Res.* (2011) 71:5588–97. 10.1158/0008-5472.Can-10-4252 21742773 PMC3379538

[48] van der Ende M Grefte S Plas R Meijerink J Witkamp RF Keijer J Mitochondrial dynamics in cancer-induced cachexia. *Biochim Biophys Acta Rev Cancer.* (2018) 1870:137–50. 10.1016/j.bbcan.2018.07.008 30059724

[49] Dave DT Patel BM. Mitochondrial metabolism in cancer cachexia: novel drug target. *Curr Drug Metab.* (2019) 20:1141–53. 10.2174/1389200220666190816162658 31418657

[50] Palomer X Alvarez-Guardia D Rodríguez-Calvo R Coll T Laguna JC Davidson MM TNF-alpha reduces PGC-1alpha expression through NF-kappaB and p38 MAPK leading to increased glucose oxidation in a human cardiac cell model. *Cardiovasc Res.* (2009) 81:703–12. 10.1093/cvr/cvn327 19038972

[51] White JP Puppa MJ Sato S Gao S Price RL Baynes JW IL-6 regulation on skeletal muscle mitochondrial remodeling during cancer cachexia in the ApcMin/+ mouse. *Skelet Muscle.* (2012) 2:14. 10.1186/2044-5040-2-14 22769563 PMC3431229

[52] Adhihetty PJ Uguccioni G Leick L Hidalgo J Pilegaard H Hood DA. The role of PGC-1alpha on mitochondrial function and apoptotic susceptibility in muscle. *Am J Physiol Cell Physiol.* (2009) 297:C217–25. 10.1152/ajpcell.00070.2009 19439529

[53] Kumar S Jiang MS Adams JL Lee JC. Pyridinylimidazole compound SB 203580 inhibits the activity but not the activation of p38 mitogen-activated protein kinase. *Biochem Biophys Res Commun.* (1999) 263:825–31. 10.1006/bbrc.1999.1454 10512765

[54] Li YP Chen Y John J Moylan J Jin B Mann DL TNF-alpha acts via p38 MAPK to stimulate expression of the ubiquitin ligase atrogin1/MAFbx in skeletal muscle. *FASEB J.* (2005) 19:362–70. 10.1096/fj.04-2364com 15746179 PMC3099533

[55] Puppa MJ Gao S Narsale AA Carson JA. Skeletal muscle glycoprotein 130’s role in Lewis lung carcinoma-induced cachexia. *FASEB J.* (2014) 28:998–1009. 10.1096/fj.13-240580 24145720 PMC3898653

