# Supplementary material for: Baoyuan Jiedu Decoction Alleviates Cancer-Induced Myotube Atrophy by Regulating Mitochondrial Dynamics Through p38 MAPK/PGC-1α Signaling Pathway
Source: Front Oncol. 2020 Sep 30;10:523577. doi: 10.3389/fonc.2020.523577 (PMC7556243; doi:10.3389/fonc.2020.523577)
Supplement: Supplementary file 1 [file Table_1.docx]

Supplementary Table 1. Detailed information of the crude drugs composed in BJD

| No | Composition of Drugs | Dose | Origin of medicinal materials | Herbal medicine processing plant | Voucher Specimens Reserve |
| --- | --- | --- | --- | --- | --- |
| BJD6-A | *Panax ginseng* C.A.Mey. | 9g | Beigang Town,  Fusong County,  Jilin Province  W:127.28°  N:42.33° | Anhui Province Xiehe pharmaceutical Yinpian Co., Ltd.  No. 2008, Yaodu Road, Qiaocheng District, Bozhou City, Anhui Province  W:115.78°  N:33.85° | Department of pharmacy, Clinic Department of Zhejiang Chinese Medical University |
| BJD6-B | *Aconitum carmichaelii* Debx. | 9g | Taiping Town,  Jiangyou City,  Sichuan Province  W:104.75°  N:31.78° | Anhui Province Xiehe pharmaceutical Yinpian Co., Ltd.  No. 2008, Yaodu Road, Qiaocheng District, Bozhou City, Anhui Province  W:115.78°  N:33.85° | Department of pharmacy, Clinic Department of Zhejiang Chinese Medical University |
| BJD6-C | *Astragalus mongholicus* Bunge. | 18g | Taiping Town,  Jiangyou City,  Sichuan Province  W:104.75°  N:31.78° | Anhui Province Xiehe pharmaceutical Yinpian Co., Ltd.  No. 2008, Yaodu Road, Qiaocheng District, Bozhou City, Anhui Province  W:115.78°  N:33.85° | Department of pharmacy, Clinic Department of Zhejiang Chinese Medical University |
| BJD6-D | *Angelica sinensis* (Oliv.) Diels. | 15g | Meichuan Town,  Min county，  Gansu Province  W:104.04°  N:34.41° | Anhui Province Xiehe pharmaceutical Yinpian Co., Ltd.  No. 2008, Yaodu Road, Qiaocheng District, Bozhou City, Anhui Province  W:115.78°  N:33.85° | Department of pharmacy, Clinic Department of Zhejiang Chinese Medical University |
| BJD6-E | *Lonicera japonica* Thunb. | 12g | Meichuan Town,  Min county，  Gansu Province  W:104.04°  N:34.41° | Anhui Province Xiehe pharmaceutical Yinpian Co., Ltd.  No. 2008, Yaodu Road, Qiaocheng District, Bozhou City, Anhui Province  W:115.78°  N:33.85° | Department of pharmacy, Clinic Department of Zhejiang Chinese Medical University |
| BJD6-F | *Glycyrrhiza uralensis* Fisch. ex DC | 6g | Meichuan Town,  Min county，  Gansu Province  W:104.04°  N:34.41° | Anhui Province Xiehe pharmaceutical Yinpian Co., Ltd.  No. 2008, Yaodu Road, Qiaocheng District, Bozhou City, Anhui Province  W:115.78°  N:33.85° | Department of pharmacy, Clinic Department of Zhejiang Chinese Medical University |
