# Supplementary material for: Baoyuan Jiedu Decoction Alleviates Cancer-Induced Myotube Atrophy by Regulating Mitochondrial Dynamics Through p38 MAPK/PGC-1α Signaling Pathway
Source: Front Oncol. 2020 Sep 30;10:523577. doi: 10.3389/fonc.2020.523577 (PMC7556243; doi:10.3389/fonc.2020.523577)
Supplement: Supplementary file 2 [file Data_Sheet_1.PDF]

<table>
<tr><th colspan="2">Original gel images</th><th>Position in the manuscript</th></tr>
<tr><td colspan="2"></td><td>Figure 7 E</td></tr>
<tr><td>①
②</td><td>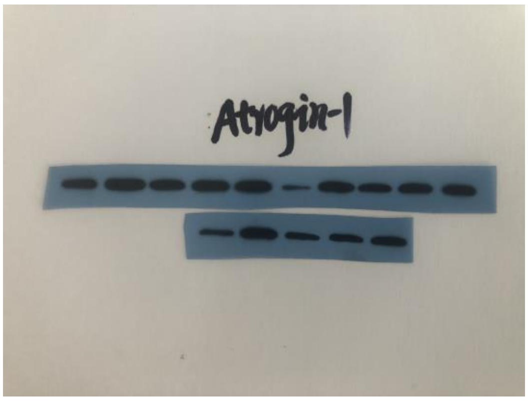

Atrogin-1</td><td>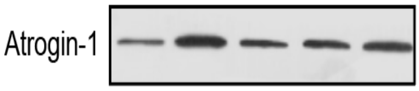

Remarks: the left image ② was used for Atrogin-1 in the manuscript.</td></tr>
<tr><td>①
②</td><td>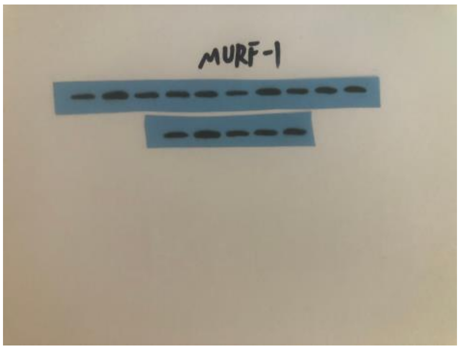

MuRF-1</td><td>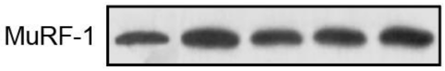

Remarks: the left image ② was used for MuRF-1 in the manuscript.</td></tr>
<tr><td>①
②</td><td>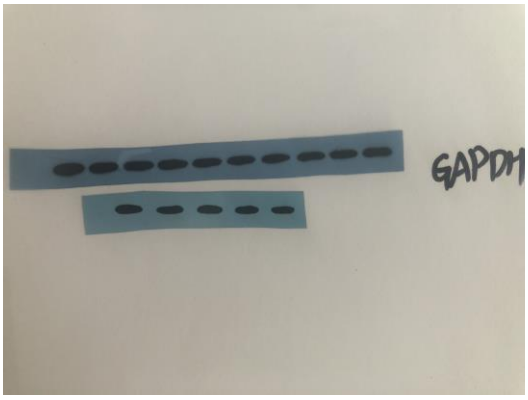

GAPDH</td><td>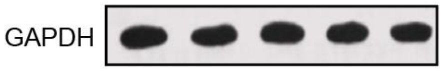

Remarks: the left image ② was used for GAPDH in the manuscript.</td></tr>
<tr><td colspan="2"></td><td>Figure 7 F</td></tr>
<tr><td>①
②</td><td></td><td>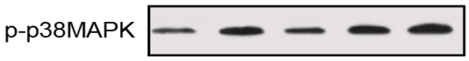

Remarks: the left image ① was used for p-p38 MAPK in the manuscript.</td></tr>
</table>

③	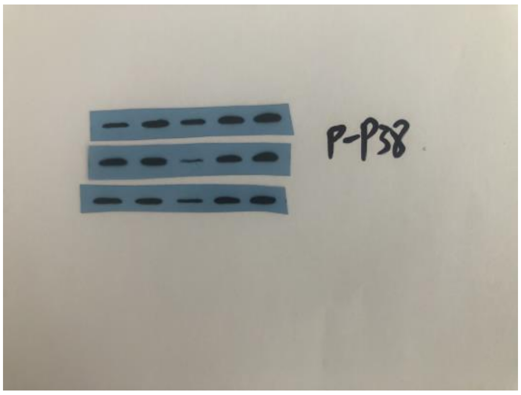<br>p-p38 MAPK	
①<br>②<br>③	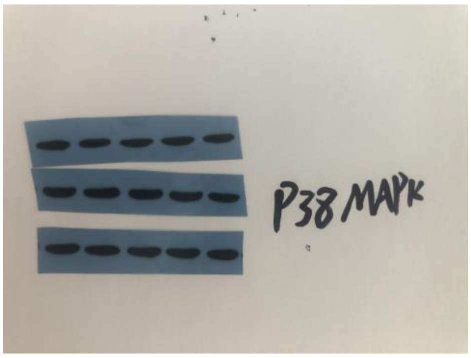<br>p38 MAPK	p38MAPK 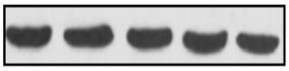<br>Remarks: the left image ② was used for p38 MAPK in the manuscript.
①<br>②<br>③	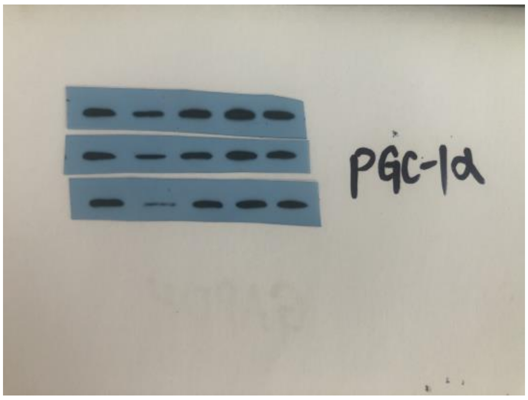<br>PGC-1α	PGC-1α 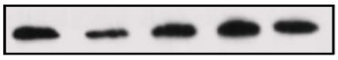<br>Remarks: the left image ① was used for PGC-1α in the manuscript.
①<br>②<br>③	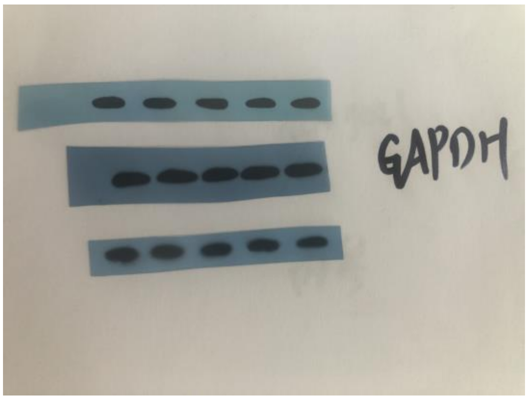<br>GAPDH	GAPDH 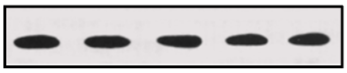<br>Remarks: the left image ① was used for GAPDH in the manuscript.
