# Supplementary material for: Baoyuan Jiedu Decoction Alleviates Cancer-Induced Myotube Atrophy by Regulating Mitochondrial Dynamics Through p38 MAPK/PGC-1α Signaling Pathway
Source: Front Oncol. 2020 Sep 30;10:523577. doi: 10.3389/fonc.2020.523577 (PMC7556243; doi:10.3389/fonc.2020.523577)
Supplement: Supplementary file 3 [file Data_Sheet_2.PDF]

	**Original gel images**	**Position in the manuscript**
		**Figure 2E**
①<br>②<br>③	1 2 3 4 5 6 7<br>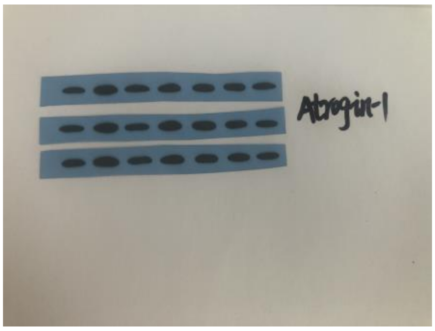<br>Atrogin-1	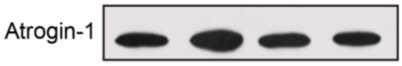<br>Remarks: The 1, 2, 3, 7 of the left image ② was used for Atrogin-1 in the manuscript.
①<br>②<br>③	1 2 3 4 5 6 7<br>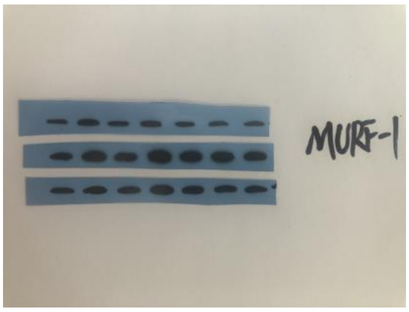<br>MuRF-1	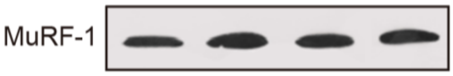<br>Remarks: The 1, 2, 3, 7 of the left image ③ was used for MuRF-1 in the manuscript.
①<br>②<br>③	1 2 3 4 5 6 7<br>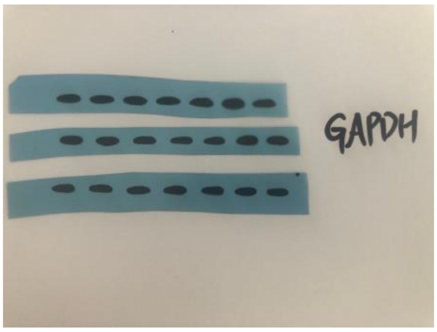<br>GAPDH	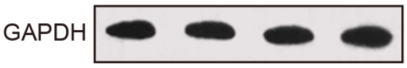<br>Remarks: The 1, 2, 3, 4 of the left image ③ was used for GAPDH in the manuscript. We found that there is a mistake image in the manuscript, it is requested for correction.
		**Figure 4G**
①<br>②<br>③	1 2 3 4 5 6 7<br>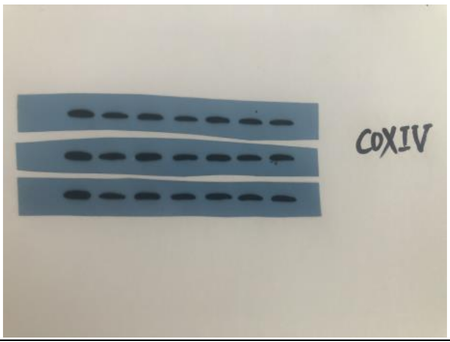	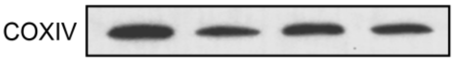<br>Remarks: The 1, 2, 3, 4 of the left image ③ was used for COXIV in the manuscript.

	COXIV	
①<br>②<br>③	1 2 3 4 5 6 7<br>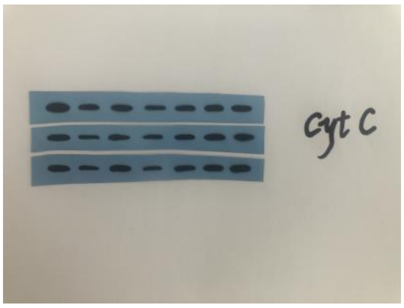<br><br>Cyt C	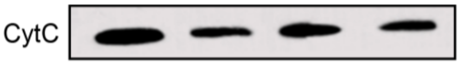<br><br>Remarks: The 1, 2, 3, 4 of the left image ② was used for Cyt C in the manuscript.
①<br>②<br>③	1 2 3 4 5 6 7<br>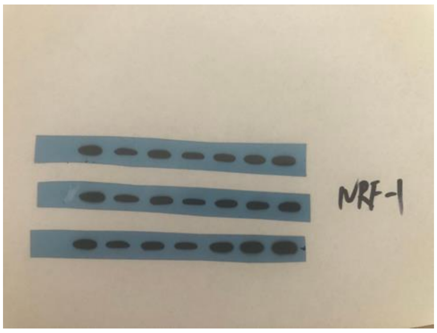<br><br>NRF-1	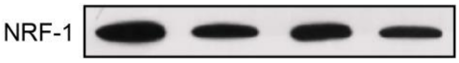<br><br>Remarks: The 1, 2, 3, 4 of the left image ② was used for NRF-1 in the manuscript.
①<br>②<br>③	1 2 3 4 5 6 7<br>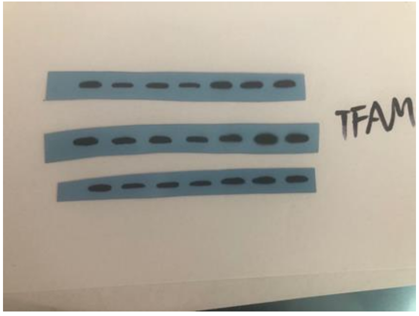<br><br>TFAM	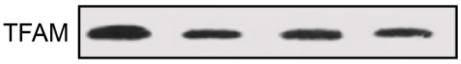<br><br>Remarks: The 1, 2, 3, 4 of the left image ① was used for TFAM in the manuscript.
①<br>②<br>③	1 2 3 4 5 6 7<br>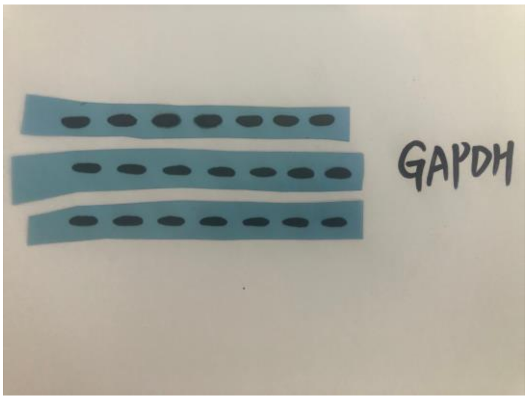<br><br>GAPDH	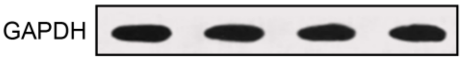<br><br>Remarks: The 1, 2, 3, 4 of the left image ② was used for GAPDH in the manuscript.

		**Figure 5C**
①<br>②<br>③	1 2 3 4 5 6 7<br>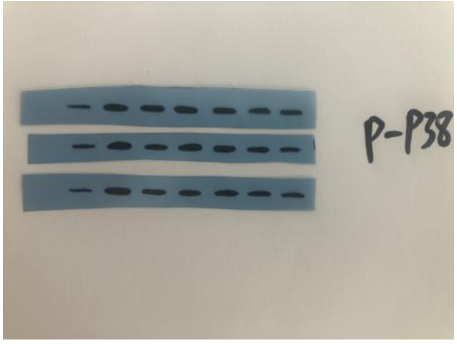<br>p-p38 MAPK	p-p38 MAPK 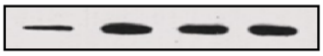<br>Remarks: The 1, 2, 3, 4 of the left image ① was used for p-p38 MAPK in the manuscript.
①<br>②<br>③	1 2 3 4 5 6 7<br>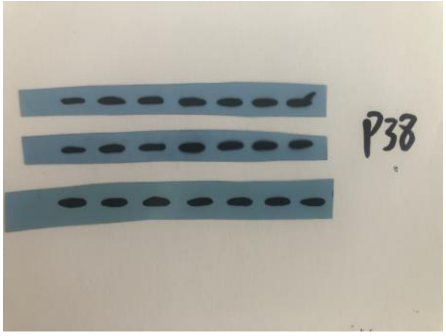<br>p38 MAPK	p38 MAPK 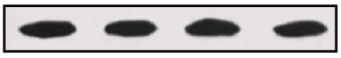<br>Remarks: The 1, 2, 3, 4 of the left image ③ was used for p38 MAPK in the manuscript.
①<br>②<br>③	1 2 3 4 5 6 7<br>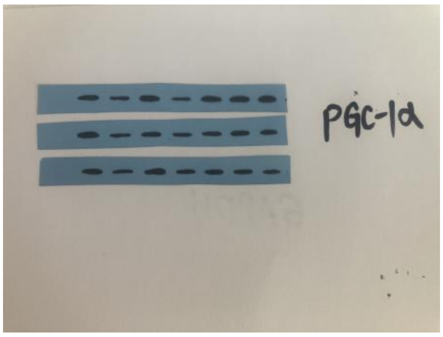<br>PGC-1α	PGC-1α 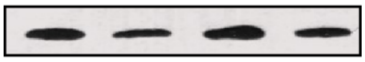<br>Remarks: The 1, 2, 3, 4 of the left image ③ was used for PGC-1α in the manuscript.
①<br>②<br>③	1 2 3 4 5 6 7<br>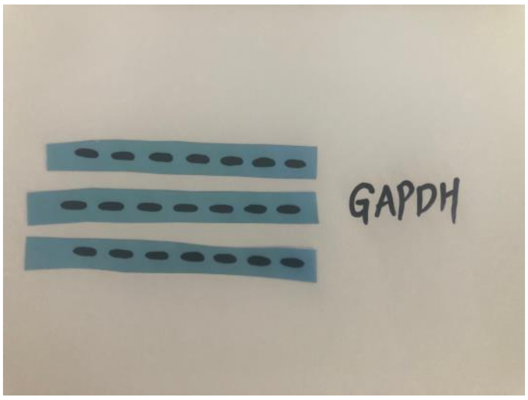	GAPDH 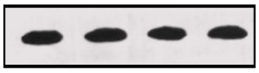<br>Remarks: The 1, 2, 3, 4 of the left image ① was used for GAPDH in the manuscript.

	GAPDH	
